# Novel insight into nicotinamide adenine dinucleotide and related metabolites in cancer patients undergoing surgery

**DOI:** 10.1038/s41598-024-66004-1

**Published:** 2024-07-17

**Authors:** Hiroaki Fujita, Taiichi Wakiya, Yota Tatara, Keinosuke Ishido, Yoshiyuki Sakamoto, Norihisa Kimura, Hajime Morohashi, Takuya Miura, Takahiro Muroya, Harue Akasaka, Hiroshi Yokoyama, Taishu Kanda, Shunsuke Kubota, Aika Ichisawa, Kenta Ogasawara, Daisuke Kuwata, Yoshiya Takahashi, Akie Nakamura, Keisuke Yamazaki, Takahiro Yamada, Ryo Matsuyama, Masanobu Kanou, Kei Yamana, Ken Itoh, Kenichi Hakamada

**Affiliations:** 1 Department of Gastroenterological Surgery, Hirosaki University Graduate School of Medicine, 5 Zaifu-Cho, Hirosaki City, Aomori 036-8562 Japan; 2 Department of Stress Response Science, Center for Advanced Medical Research, Hirosaki University Graduate School of Medicine, Hirosaki, Aomori Japan; 3 Department of Surgery, Jichi Medical University, Shimotsuke, Tochigi Japan; 4 Nutraceutical Group, New Business Development Unit, Teijin Limited, Hino, Tokyo Japan; 5 Discovery DMPK Research Group, Toxicology & DMPK Research Department, Teijin Institute for Bio-Medical Research, Teijin Pharma Limited, Hino, Tokyo Japan; 6 NOMON Co. Ltd., Kasumigaseki, Chiyoda-Ku, Tokyo Japan

**Keywords:** Cancer, Nicotinamide adenine dinucleotide, Nicotinamide mononucleotide, Surgery, Precision medicine, Metabolism, Biophysical chemistry, Enzymes, Metabolomics, Cancer metabolism, Biochemistry, Cancer, Biogeochemistry, Oncology, Surgical oncology

## Abstract

Nicotinamide adenine dinucleotide (NAD +) plays a pivotal role in numerous cellular functions. Reduced NAD + levels are postulated to be associated with cancer. As interest in understanding NAD + dynamics in cancer patients with therapeutic applications in mind grows, there remains a shortage of comprehensive data. This study delves into NAD + dynamics in patients undergoing surgery for different digestive system cancers. This prospective study enrolled 99 patients with eight different cancers. Fasting blood samples were obtained during the perioperative period. The concentrations of NAD + , nicotinamide mononucleotide (NMN), and nicotinamide riboside were analyzed using tandem mass spectrometry. After erythrocyte volume adjustment, NAD + remained relatively stable after surgery. Meanwhile, NMN decreased the day after surgery and displayed a recovery trend. Interestingly, liver and pancreatic cancer patients exhibited poor postoperative NMN recovery, suggesting a potential cancer type-specific influence on NAD + metabolism. This study illuminated the behavior of NAD + in surgically treated cancer patients. We identified which cancer types have particularly low levels and at what point depletion occurs during the perioperative period. These insights suggest the need for personalized NAD + supplementation strategies, calibrated to individual patient needs and treatment timelines. **Clinical trial registration** jRCT1020210066.

## Introduction

Nicotinamide adenine dinucleotide (NAD^+^) is an essential metabolite for maintaining cellular homeostasis. Multiple reduction–oxidation (redox) metabolic reactions for energy production are based on NAD^+^. Moreover, NAD^+^ is essential for non-redox NAD-dependent enzymes, including sirtuins (SIRT), poly (adenosine diphosphate-ribose) polymerases (PARP), and cyclic adenosine diphosphate-ribose synthetases. NAD^+^ influences many signaling pathways, including metabolic pathways, DNA repair, chromatin remodeling, posttranslational modifications, immune responses, apoptosis, and cellular senescence. Hence, decreased NAD^+^ levels can lead to the deterioration of these functions and their associated diseases^1–4^.

Declining NAD^+^ levels are linked to aging^5,6^. This evidence suggests that we all are at risk for defects in NAD-related function as we age, resulting in aging-related diseases^7^. One of the most typical aging-related diseases is cancer^8–12^. Several lines of evidence have demonstrated that DNA repair capacity decreases with aging^13–16^. Defects in DNA repair increase cancer development^16,17^.

Cancer cells increase NAD^+^ levels to satisfy their high glycolytic demands for uncontrolled proliferation^1,18^. Paradoxically, considering the limited energy resources in the tumor microenvironment, NAD^+^ in non-cancer cells may become depleted. In addition, various types of cancer treatments, including radiotherapy and chemotherapy, can affect DNA repair in both cancer cells and normal cells, resulting in increased consumption of NAD^+^^19,20^. Based on this information, it is theoretically speculated that NAD^+^ levels tend to be depleted in cancer patients, especially patients of advanced age. Unfortunately, it is also easy to imagine that NAD^+^ decline would reduce anti-tumor immunity, which is favorable to cancer growth.

Nicotinamide mononucleotide (NMN) supplementation is a promising strategy to tackle the adverse condition caused by NAD^+^ depletion. The main NAD^+^ synthesis pathway is via the salvage pathway, in which the NAD^+^ catalysis, through a number of enzymes (PARP, SIRT, CD38, etc.), generates nicotinamide, which is recycled into NMN by nicotinamide phosphoribosyl transferase (NAMPT) and adenylated to NAD^+^ by nicotinamide mononucleotide adenylyl transferases^21^. Thus, NMN is an orally bioavailable precursor to NAD^+^. A promising randomized, placebo-controlled, double-blind trial to evaluate the effects of NMN supplementation on metabolic function in postmenopausal women proved that NMN supplementation led to a significant increase in the NAD^+^ level in plasma and peripheral blood mononuclear cells^22^. This randomized trial also demonstrated that NMN supplementation increases muscle insulin sensitivity, implying it improves biological function in humans^22^. In addition, several recent clinical trials have shown that oral NMN supplementation improves various functions, including physical performance, sleep quality, and muscle function, in healthy adults^23,24^.

These results increasingly have evoked the notion among researchers that NAD^+^ metabolism-related pathologies could lead to new therapeutic strategies for cancer patients. Meanwhile, conversely, an excessive increase may contribute to cancer growth^1^. In short, to establish a novel therapeutic strategy using NMN supplementation, NAD^+^ must be kept within the optimal range after intervention in cancer patients. However, basic data on NAD^+^ and related metabolites in cancer patients is definitely insufficient. Therefore, we conducted a prospective cross-sectional study to clarify the levels of NAD^+^ and related metabolites in patient with various types of cancer. Furthermore, we aimed to evaluate the association between NAD^+^-related factors and perioperative clinical factors. Herein, we present the first comprehensive analysis of NAD^+^ and related metabolites in surgically treated patients with eight different cancers of the digestive system.

## Results

### Comparison of the perioperative characteristics among patients with different cancer types

Of the 99 cancer patients recruited, 93 (93.9%) were included in the study. The clinical characteristics of the patients are shown in Table 1. Appropriate surgery was ultimately performed on 74 of them (79.6%). Operative and postoperative outcomes are also shown in Table 1. Ten patients were treated with preoperative chemotherapy. During this study period, no patients were treated with preoperative radiotherapy, adjuvant chemotherapy, or PARP inhibitors.

**Table 1:** Comparison of perioperative characteristics for the entire cohort.

	All (n = 93)	Esophagus (n = 6)	Stomach (n = 17)	Bile duct (n = 13)	Liver (n = 5)	Pancreas (n = 14)	Colorectum (n = 36)	Meta liver (n = 2)
Gender, male	56 (60.2)	5 (83.3)	12 (70.6)	7 (53.8)	4 (80.0)	7 (50.0)	19 (52.8)	2 (100.0)
Age, year	72 (40–93)	62 (44–73)	70 (52–79)	69 (55–78)	81 (70–84)	75 (62–81)	73 (40–93)	59 (53–64)
BMI, kg/m^2^	22.1 (15.4–30.4)	24.7 (17.9–27.5)	22.6 (16.4–29.9)	22.8 (18.7–28.2)	25.4 (21.6–27.4)	21.7 (17.7–25.9)	21.1 (15.4–30.4)	21.8 (21.1–22.6)
Albumin, g/dL	4.0 (2.6–4.9)	4.0 (3.8–4.6)	4.2 (2.6–4.9)	3.9 (2.9–4.3)	3.8 (2.9–4.3)	3.8 (2.0–4.5)	4.0 (2.9–4.6)	4.6 (4.3–4.8)
Resected case, n	74 (79.6)	4 (66.6)	16 (94.1)	11 (84.6)	4 (80.0)	7 (50.0)	32 (88.9)	0
Preoperative therapy, n	12 (12.9)	4 (66.6)	0	0	0	3 (42.9)	5 (15.6)	-
Duration from initial presentation to admission, day	33 (1–165)	83 (5–106)	25 (2–67)	43 (18–80)	25 (17–43)	49 (30–99)	23 (1–165)	-
Operation time, min	259 (63–701)	577 (540–608)	253 (162–352)	346 (129–587)	160 (134–218)	379 (212–701)	229 (63–701)	-
Intraoperative blood loss, mL	80 (0–1448)	540 (50–825)	80 (0–770)	983 (150–2775)	1028 (290–1350)	240 (70–270)	20 (0–900)	-
Intraoperative ABT, n	0	0	0	0	0	0	0	-
Postoperative complications (Clavien-Dindo classification grade ≥ 3), n	10	0	3	2	0	1	4	-
Postoperative hospital stay, day	11 (3–89)	13 (12–19)	10 (7–65)	12 (3–89)	10 (9–12)	13 (11–36)	10 (7–27)	-

ABT, allogeneic blood transfusion; ALT, alanine aminotransferase; ANH, acute normovolemic hemodilution; ASA-PS, American Society of Anesthesiologists physical status; AST, aspartate aminotransferase; BMI, body mass index; CA19-9, carbohydrate antigen 19–9; CEA, carcinoembryonic antigen; CRP, C-reactive protein; STD, standard management; UICC, Union for International Cancer Control.

^†^: All of the patients were diagnosed with M1 due to positive lymph nodes other than the regional lymph nodes.

### Concentrations of NAD^+^ and related metabolites among the entire subject base

First, we assessed the concentrations of NAD^+^ and related metabolites. Since plasma NAD^+^ concentrations were below the limit of quantification^25^, and peripheral blood mononuclear cells contained less than 5% of whole blood NAD^+^ (data not shown), whole blood NAD^+^ was considered to be derived almost entirely from red blood cell (RBC). In addition, because RBC counts and volume vary with age, sex, and surgical bleeding, NAD^+^ values in this study were adjusted for RBC counts and mean corpuscular volume (MCV), i.e., RBC count was multiplied by MCV to calculate total RBC volume, and the concentration in whole blood was converted to concentration in total RBC volume, which is the RBC volume-adjusted concentration.

**Figure 1:**
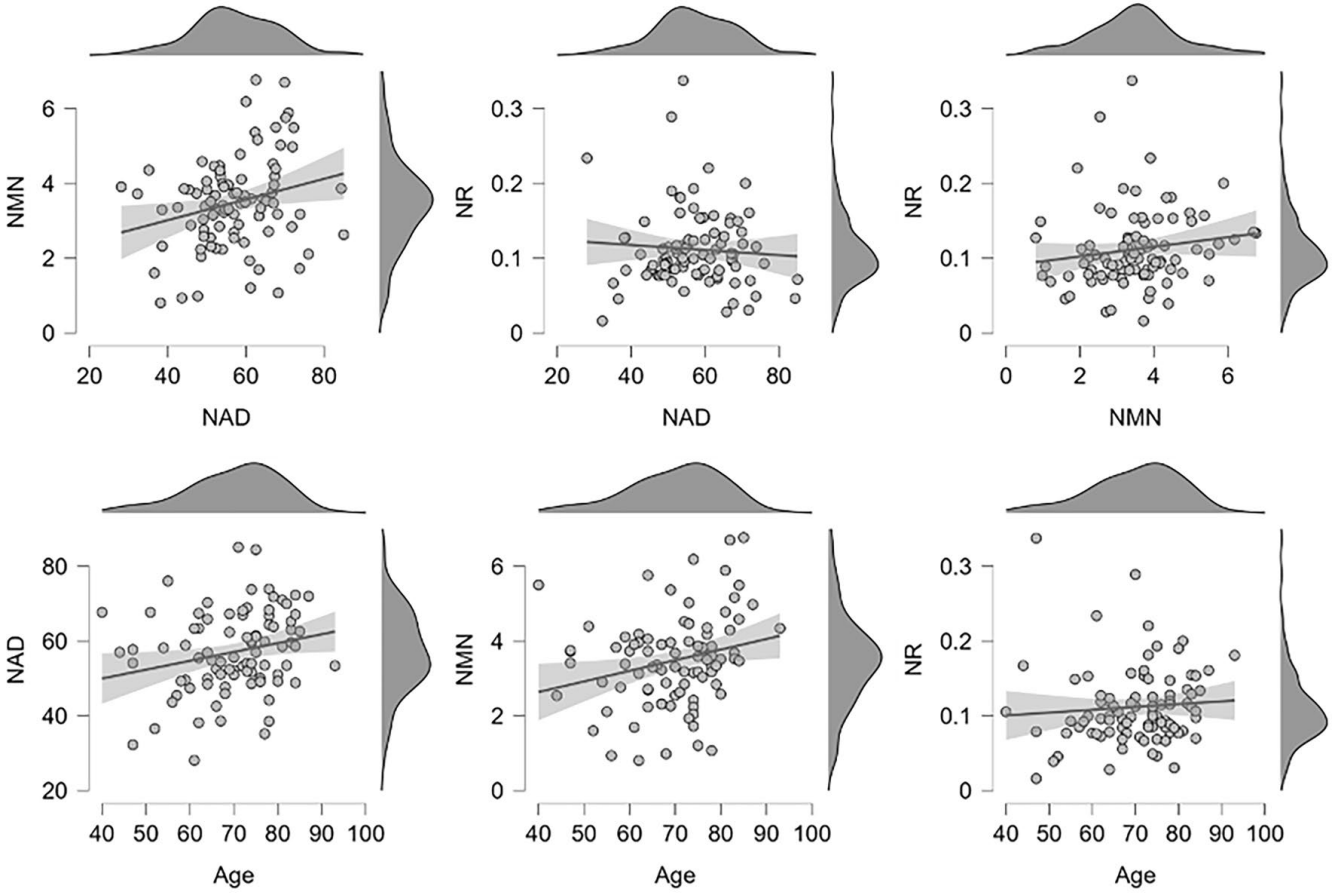
Scatter plot with correlation and density plots for age, RBC volume-corrected NAD^+^ (µM), NMN (µM), and NR (µM) from initial medical examination samples. Linear regression lines and 95% confidence intervals are represented. The quantified whole blood NAD^+^ value was represented as a concentration adjusted for RBC, which is MCV multiplied by the number of RBCs.

The mean concentrations of NAD^+^, NMN, and NR measured from the whole blood of these cancer patients at their initial presentation were 22.00 µM, 1.34 µM, and 0.04 µM, respectively (Table 2). NAD^+^ concentrations were 16 times higher than the NMN concentrations. The concentrations of NAD^+^, NMN, and NR corrected for RBC volume were 57.06 µM, 3.49 µM, and 0.11 µM, respectively (Table 2). Red blood cell counts were higher in males than in females, but there were no significant differences in whole blood NAD^+^ (Supplemental Fig. S1). NAD^+^ values corrected for RBC volume were also not significantly different (Supplemental Fig. S1). We then evaluated a correlation among RBC volume-corrected NAD^+^, NMN, NR, and age (Fig. 1). Pearson's correlation analysis showed a positive correlation between NAD^+^ and NMN (r = 0.246, *p* = 0.020). Since NMN is a metabolic precursor of NAD^+^, a positive correlation between NAD^+^ and NMN may have been observed in this study because it measured NAD^+^-related metabolites mainly in erythrocytes. A weak positive correlation between age and NAD^+^ was found (r = 0.230, *p* = 0.029), but the narrow age range in this study does not allow conclusions to be drawn about the relationship between aging and blood NAD^+^.

**Table 2:** Descriptive statistics of NAD^+^, NMN, and NR measured for cancer patients.

	N	Mean	SD	Median	Min	Max
Age	91	69.8	10.69	72	40	93
RBC (10^6^ cells/µL)	91	4.26	0.59	4.35	2.91	5.91
MCV (fL)	90	91.75	6.48	92.45	69.1	104.5
Concentration in whole blood						
NAD^+^ (µM)	91	22.00	4.37	21.66	9.8	31.85
NMN (µM)	91	1.34	0.44	1.35	0.35	2.4
NR (µM)	91	0.04	0.02	0.04	0.01	0.15
Concentration in RBCs						
NAD^+^ (µM)	90	57.06	10.9	56.91	28.08	85.03
NMN (µM)	90	3.49	1.23	3.48	0.8	6.76
NR (µM)	90	0.11	0.05	0.1	0.02	0.34

MCV, mean corpuscular volume; NAD^+^, nicotinamide adenine dinucleotide; NMN, nicotinamide mononucleotide; NR, nicotinamide riboside chloride; RBC, red blood cell.

### Chronological changes in NAD^+^ and related metabolites

**Figure 2:**
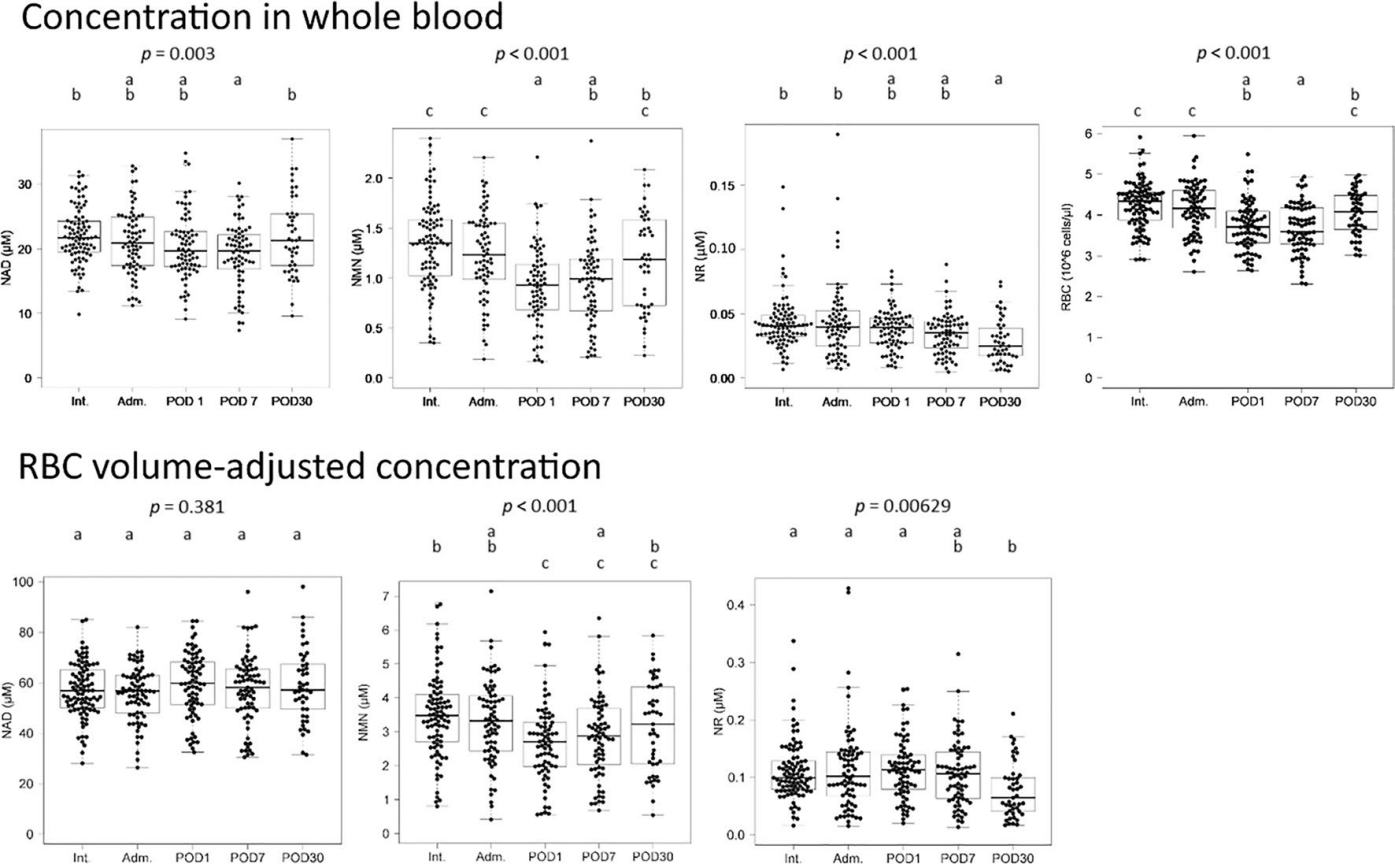
Boxplots for NAD^+^, NMN, and NR measurements of whole blood samples from cancer patients. Each measurement was represented as a concentration in whole blood or a concentration adjusted for RBCs volume. Blood samples were collected at Int., Adm., POD1, POD7, and POD30. Significant differences among groups were determined by ANOVA analysis. The letters above the boxplot indicate significant differences between the means of each group (Tukey's post-hoc test). One group is significantly different from another if there is no common letter between the two groups. Adm., admission; Int., initial intake medical examination; POD1, the day after surgery; POD7, 1 week after surgery; POD30, 1 month after surgery.

We assessed how NAD^+^ and related metabolites change perioperatively (Fig. 2). Whole blood NAD^+^ and NMN showed a significant decrease on postoperative day (POD) 1. Since NR showed values near the limit of quantification, no noticeable changes of significance could be found. RBC counts also decreased significantly postoperatively, suggesting that the decrease in whole blood NAD^+^ and NMN was due to bleeding during surgery. When the quantitative values were corrected for RBC volume, it was found that NAD^+^ did not change during the perioperative period, indicating that NAD^+^ was maintained at a constant level. Meanwhile, NMN corrected for RBC volume decreased on POD1. Subsequently, NMN concentrations showed a recovery tendency between POD1 and POD30.

### Before and after surgery comparisons for each patient

**Figure 3:**
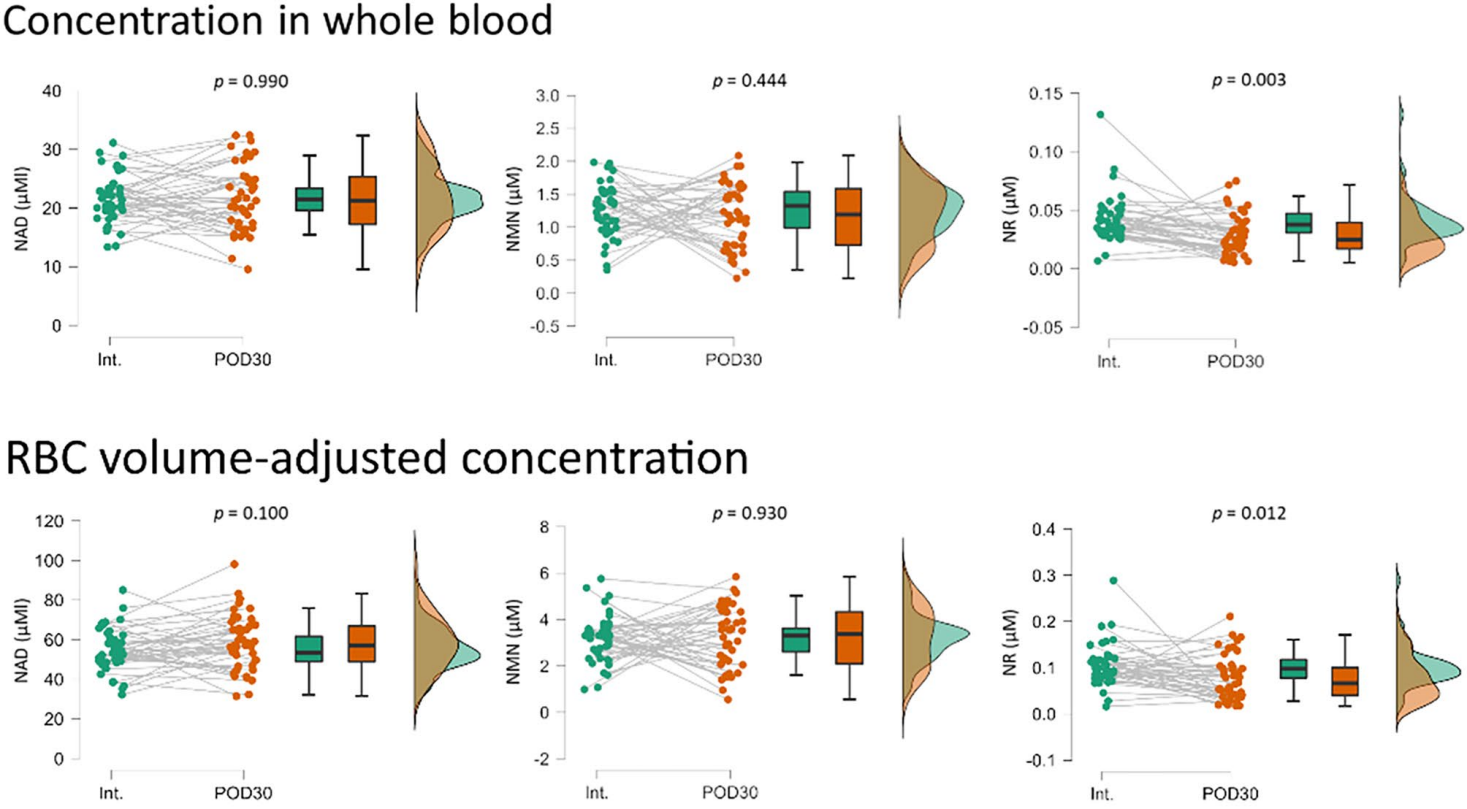
Raincloud plots of NAD^+^, NMN, and NR measurements for whole blood samples from cancer patients at Int. and POD30. Each measurement was represented as a concentration in whole blood or a concentration adjusted for RBC volume. Significant differences between groups were determined using a paired t test. Int., initial intake medical examination; POD30, 1 month after surgery.

**Figure 4:**
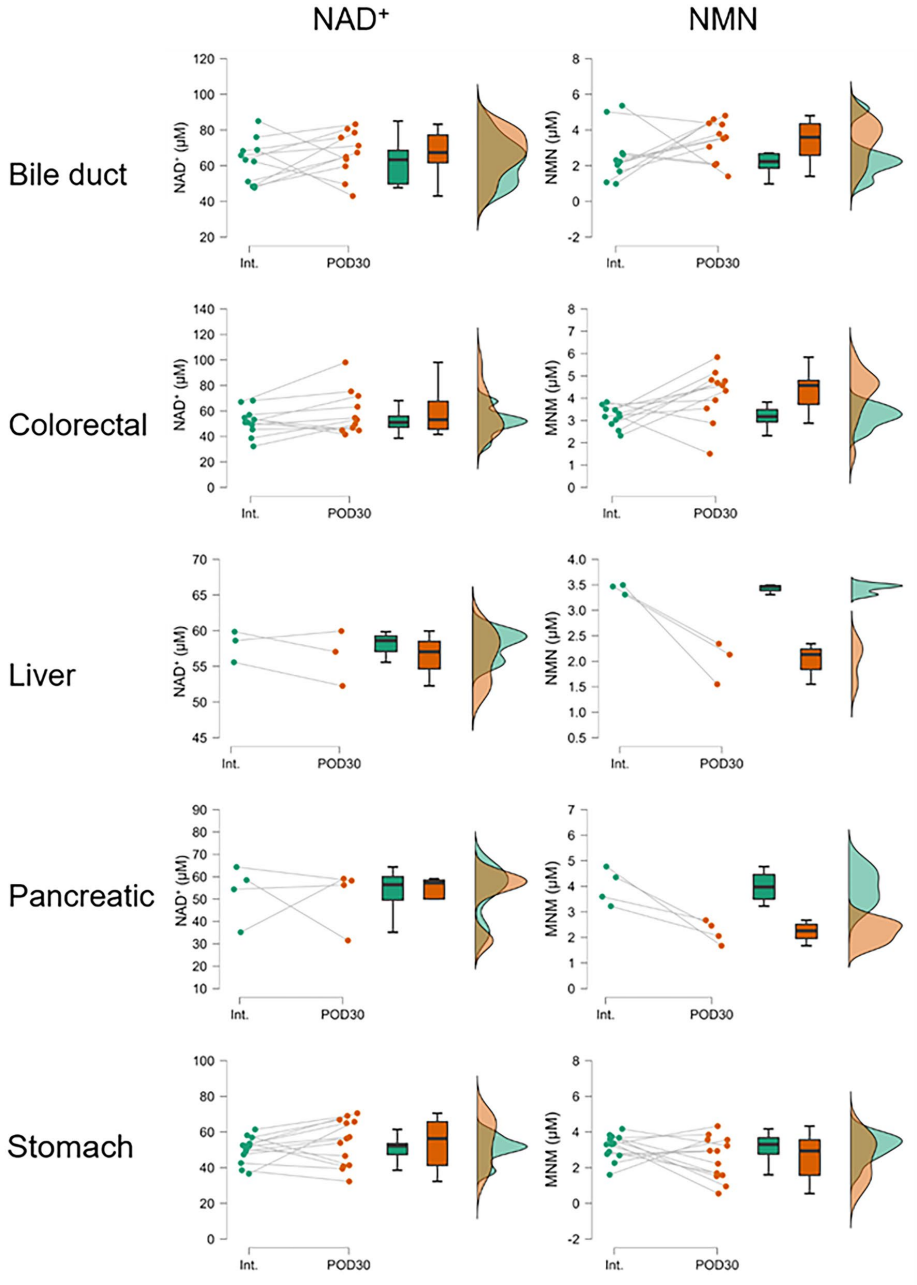
Rain cloud plots of NAD^+^ and NMN measurements of whole blood samples stratified by cancer patients at Int. and POD30. Each measurement was represented as RBC volume-adjusted concentrations. Significant differences between groups were determined using a paired t test. Int., initial intake medical examination; POD30, 1 month after surgery.

A paired t test was used to test whether NAD^+^ and related metabolites had changed in each patient at Int. and POD30 (Fig. 3). As a result, it was discovered that whole blood and RBC volume-corrected NAD^+^ and NMN were not affected by cancer resection. However, RBC volume-corrected NMN seemed to partition into two patterns, with some samples showing a decrease at POD30 and others showing an increase. When stratified by cancer type, colorectal cancer showed significant increases at POD30, while liver and pancreatic cancer had significantly decreases at POD30 (Fig. 4). These results indicate that the amount of NMN used to maintain NAD^+^ concentration in RBCs varies by cancer type or surgical procedure.

### Comparison among cancer types

**Figure 5:**
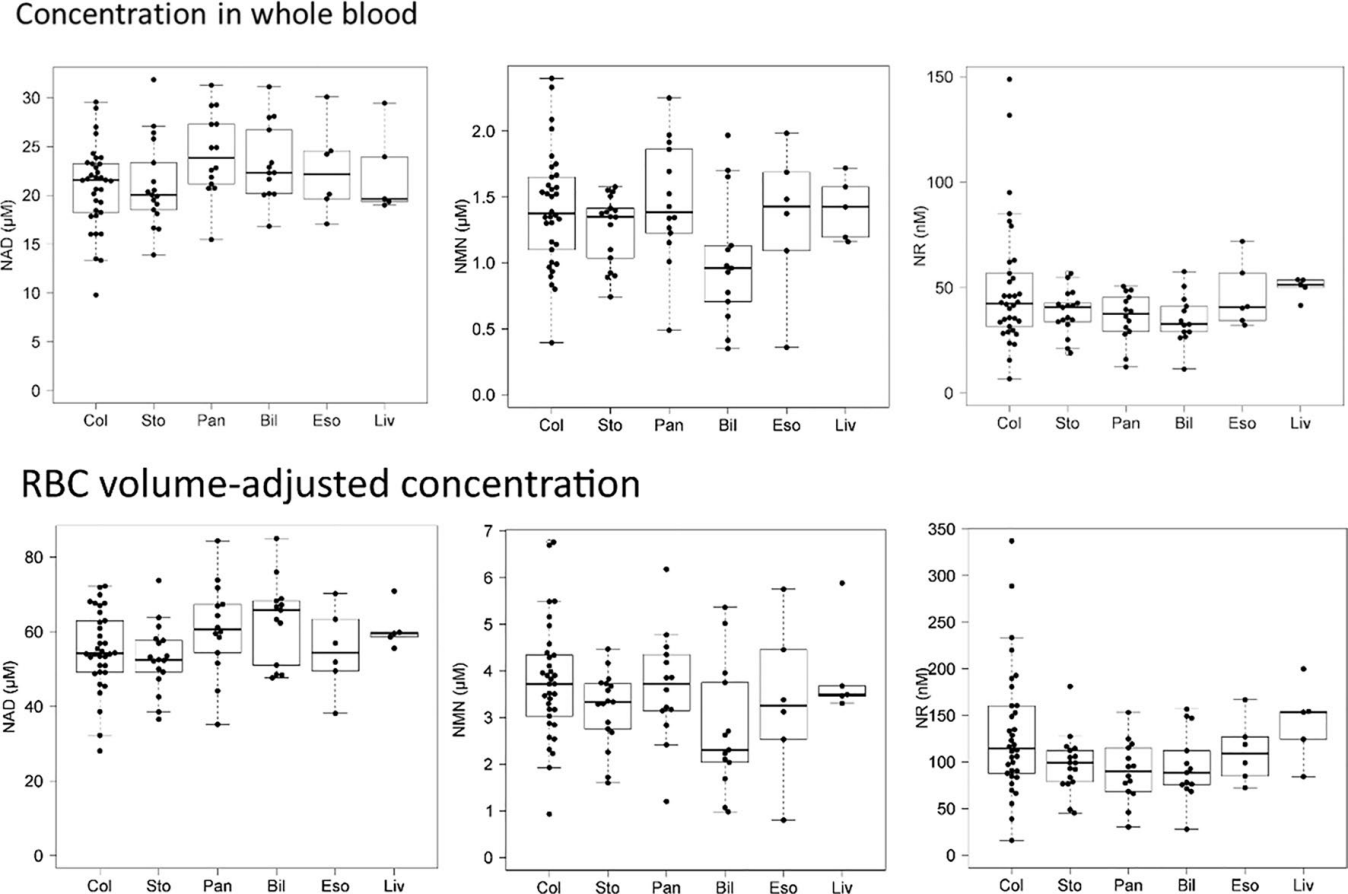
Comparison among cancer types of NAD^+^, NMN, and NR measurements for whole blood samples from cancer patients at the initial medical examination. Each measurement was represented as a concentration in whole blood or a concentration adjusted for RBC volume. Bil, bile duct; Col, colorectum; Eso, esophagus; Liv, liver; Pan, pancreas; Sto, stomach.

**Figure 6:**
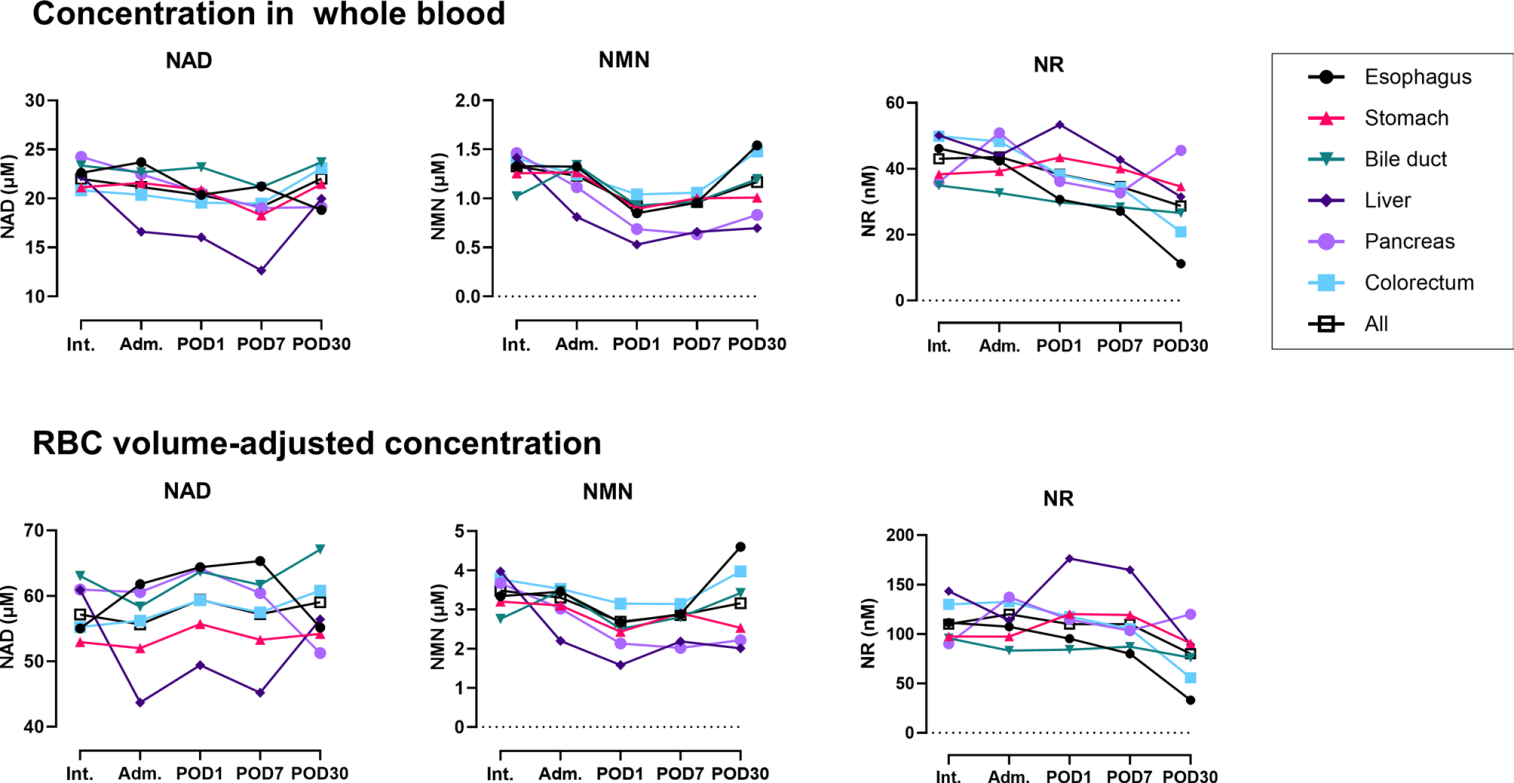
Comparison of chronological changes in NAD^+^ and related metabolites among cancer types. Each measurement was represented as a concentration in whole blood or a concentration adjusted for RBC volume. Adm., admission; Int., initial intake medical examination; POD1, the day after surgery; POD7, 1 week after surgery; POD30, 1 month after surgery.

We examined whether there were differences in NAD^+^ and related metabolites in whole blood depending on the type of cancer. As shown in Fig. 5, there were no significant differences in NAD^+^, NMN, or NR measurements between cancer types at Int. To clarify whether a chronological change in NAD^+^ and related metabolites can be different depending on the cancer type, we compared the measurements of six cancer types (Fig. 6, Supplemental Fig. S2). NAD^+^ levels decreased from POD1 to POD7 in most cancer types. From POD 7 to POD 30, the NAD^+^ levels for esophageal and pancreatic cancer decreased to below average. Perioperative NAD^+^ levels in patients with bile duct cancer were higher than average, while those in patients with gastric cancer were lower than average. The NAD^+^ and NMN concentrations of patients with liver cancer were higher than average at Int., however, clearly lower than average after admission for surgery. Although large data-sets are needed to draw definitive conclusions, these data at least suggest that NAD^+^ and related metabolites can differ across cancer types during the perioperative period.

### Relationship between NAD-related metabolites and perioperative outcomes

We analyzed the impact of preoperative chemotherapy on NAD^+^ and related metabolites. Supplemental Fig. S3 presents the comparative results for pancreatic and colorectal cancer cases, both with and without preoperative chemotherapy. These comparisons did not clearly demonstrate the effect of preoperative chemotherapy on the levels of NAD^+^ and related metabolites levels. However, given the heterogeneous nature of the chemotherapy regimens used for each patient, further investigation with a larger, more homogeneous sample is necessary to draw definitive conclusions.

We further clarified the relationship between NAD^+^ and operative and postoperative outcomes. In this cohort, a negative effect of prolonged operative time on postoperative measurements was not clearly observed (Supplemental Fig. S3). Although not statistically significant, postoperative levels of NMN, in particular, tended to be lower in the severe intraoperative bleeding group (Supplemental Fig. S4). When postoperative complications were stratified into two groups based on Clavien-Dindo stratification and examined in relation to the measurements, no statistically significant relationship was observed in this cohort (Supplemental Fig. S5).

## Discussion

This prospective study revealed basic data on NAD^+^ and related metabolites in surgically treated cancer patients. Our results demonstrated that whole blood NAD^+^ and NMN levels were significantly decreased after surgery. When adjusted by RBC volume, NMN levels decreased, while NAD^+^ levels remained constant during the perioperative period. Interestingly, this decrease of NMN was severely protracted until POD30 in liver and pancreatic cancer patients. Our results yielded a practical key insight into a novel therapeutic strategy using NMN supplementation for cancer patients.

Mature erythrocytes have fewer NAD^+^ consumers such as PARPs and SIRTs because they lack nuclei and mitochondria. Although little had been known about NAD^+^ metabolism in erythrocytes, Hikosaka et al. reported on erythrocytes from mice deficient in Nmnat3, which produces NAD^+^ from NMN in the NAD^+^ salvage pathway^26^. In the Nmnat3-deficient mice, erythrocyte NAD^+^ and NMN levels were markedly reduced while NAD^+^ levels of other tissues remained intact. This NAD^+^ decline caused the impairment of glycolysis by the inhibition of glyceraldehyde-3-phosphate dehydrogenase (GADPH) with a consequent decrease in ATP^26^. Consequently, Nmnat3 knockout mice showed hemolytic anemia due to abnormal erythrocyte morphology and increased erythrocyte destruction in the spleen. They also demonstrated that Nmnat3 is most highly expressed in the cytoplasm in mature erythrocytes, which collectively showed that the integrity of the salvage pathway in erythrocytes is important for the maintenance of erythrocytes. In the present study, postoperative NAD^+^ in erythrocytes was unchanged, but NMN was decreased especially in liver and pancreatic cancer patients (Fig. 4). The reason is not clear at present, but the decrease in NMN may result from the decreased NMN synthesis by the decreased nicotinamide incorporation from plasma, which is a substrate of iNAMPT, the rate-limiting step of the salvage pathway since no apparent stress is applied to erythrocytes that cause NAD^+^ consumption during surgery. There have been no previous reports measuring plasma nicotinamide concentrations in cancer patients, but they may differ depending on the cancer type. Change in NMN transport from plasma may also have some effects. These possibilities need further clarification in the future.

Bypassing NAMPT, the rate-limiting enzyme for NAD^+^ synthesis, with NMN may be an effective strategy to restore the NAD^+^ supply in RBCs. The association between NMN supplementation and prognosis is of particular interest, as NMN was significantly lower 30 days after surgery in pancreatic and liver cancers (Fig. 4). In erythrocytes, NAD^+^ is used primarily for ATP production in glycolysis and for conversion of methemoglobin to hemoglobin^27,28^. Since hemoglobin maintenance is directly related to oxygen-carrying functions, supplementation of erythrocyte NAD^+^ may enhance energy production and improve postoperative recovery and ultimately lead to a better prognosis in cancer patients.

 In our study, the mean NAD^+^ level among cancer patients aged 50–59 years was found to be 22.18 µM. This figure is lower compared to the 31.35 µM reported for healthy individuals in the same age group in a previous study^25^, which utilized the same measurement method. It is important to clarify that our study did not directly measure NAD^+^ levels in a healthy control group; therefore, this comparison is based solely on speculative literature values. It might be either due to the low NAD + levels in cancer patients or low NAD + levels in this specific district (i.e. northern Japan area). These possibilities should be clarified in a future analysis.

Considering that NAD^+^ levels decrease with age^5,6^, we analyzed the correlation between age and NAD^+^ metabolite levels in cancer patients; however, no clear correlation emerged. Instead, we observed a weak but positive correlation. This result suggests that NAD^+^ metabolism may vary depending on the type of cancer, and that the impact of cancer on NAD^+^ metabolism might be more significant than that of aging.

One of the strengths of this study is that we have elucidated how NAD^+^ and related metabolites are altered after the loss of both the organs themselves and their functions due to surgery. At least in the first month after the operation, the impact of organ loss appears to be limited in patients with alimentary tract cancers. In contrast, postoperative NMN levels were lower than preoperative levels in patients with liver and pancreatic cancer. Interestingly, the postoperative NMN levels of bile duct cancer patients did not decrease significantly even though approximately 30% of them underwent major hepatectomy, similar to liver cancer patients, and approximately 60% underwent pancreaticoduodenectomy, similar to pancreatic cancer patients. These data suggest that factors other than organ loss, such as the type of cancer and the condition of the organ where the cancer developed, may have an effect. In this study, we did not measure NAD^+^ and related metabolite levels in non-cancerous patients who underwent surgery. Having data from non-cancerous patients would enable a clearer discussion of the effects of the surgery itself, as well as organ loss. Therefore, further comparison is necessary between patients with benign and malignant diseases who underwent the same surgical procedures. At present, the systemic NAD^+^ metabolome is largely unknown^29,30^. Therefore, NAD^+^ dynamics after organ loss should be closely monitored for a longer period of time.

Cancer is a type of the chronic disease. Accordingly, it is speculated that NAD^+^ homeostasis may change during the long course of the disease based on clinical stage and cancer therapies. Although we clarified the changes in NAD^+^ metabolites during the perioperative period, we need to accumulate information related to the long-term course of treatment of these cancers. This data is necessary before the start of an NMN supplementation trial. We hope that this study will be a starting point to complement data beyond what we were able to collect in this study.

In conclusion, we have demonstrated the dynamics of NAD^+^ and related metabolites in cancer patients undergoing surgery. We expect that future studies with long-term follow-up will expand our understanding of the NAD^+^ metabolism of cancer patients, leading to new therapeutic strategies.

## Methods

### Ethical approval and informed consent

This single-center, prospective observational cohort study was approved by the Committee of Medical Ethics of Hirosaki University Graduate School of Medicine (Aomori, Japan; reference no. 2021-073). This study was registered with the Japan Registry of Clinical Trials (jRCT1020210066). Oral and written informed consent were obtained from each patient before enrollment. Our study did not include minors. This study was designed and carried out in accordance with the Declaration of Helsinki.

### Study participants

Between December 2021 and July 2022, 99 Japanese cancer patients were recruited for this study. The inclusion criteria were as follows: having been referred to the department for curative treatment, being age 20 or older, and providing written informed consent. The exclusion criteria can be found in Supplemental information 1.

### Study timeline

Based on institutional policies, participants received the standard treatment associated with their cancer type. Blood was collected from the forearm during the initial intake medical examination (Int.), on admission (Adm.), and on the days following surgery—specifically, 1 day after (Post-Operative Day 1, POD1), 1 week after (Post-Operative Day 7, POD7), and 1 month after (Post-Operative Day 30, POD30)—as part of routine clinical laboratory exams. Residual specimens from the laboratory exam were used for measuring NAD^+^ and related metabolites. There have been several reports of NAD^+^ measured using enzymatic methods on human whole blood samples, all of which report no circadian rhythm^31,32^. Therefore, the time of blood collection was not strictly controlled in this study, but 75% of all samples were collected between 6:00 AM and 9:00 AM. A fasting blood test was generally performed.

### Objectives

The primary objective is to clarify the levels of NAD^+^ and related metabolites in patients with various types of cancer at different times during perioperative period. The secondary objectives are to evaluate the association between NAD^+^-related factors and perioperative clinical factors. The following clinical factors were analyzed: gender, age, disease (cancer type), body mass index (BMI), history of neoadjuvant therapy, duration from initial presentation to admission for the surgery, preoperative serum albumin level, operative procedure, operative time, intraoperative blood loss volume, allogeneic red blood cell transfusion, postoperative complications based on Clavien-Dindo classification grade, and duration of postoperative hospital stay.

### Materials

β-Diphosphopyridine nucleotide was purchased from FUJIFILM Wako Pure Chemical Co. β-Nicotinamide adenine dinucleotide-d4, β-nicotinamide mononucleotide, and β-nicotinamide-d4 mononucleotide were purchased from Toronto Research Chemicals, Inc. NR and nicotinamide riboside-d4 triflate came from MedKoo Biosciences, Inc. and Santa Cruz Biotechnology, Inc., respectively.

### Sample preparation

Measuring NAD^+^ and related metabolites from whole blood was performed with minor modifications to the previous study^25^. Whole blood samples from patients were collected in INSEPACK ST vacuum tubes (Sekisui Medical) containing EDTA-2Na as the anticoagulant. From these whole blood samples, 5 µL were dropped onto QIAcard FTA DMPK-B cards (QIAGEN) to produce dried blood spots. After drying for 10 min at room temperature, the dried blood samples were sealed in a bag with a desiccant and stored at 4 °C until analysis (up to 1 week). From the cards, 8-mm diameter spots were punched out and immersed in the extraction solution (290 µL water and 10 µL internal standard solution) in capped tubes. These tubes were shaken for 30 min in a tumble-shaker. Subsequently, 120 µL of the extraction solution was added to 240 µL of acetonitrile. After centrifugation (24,250 g for 10 min at 4 °C), the supernatant was aliquoted into vials and stored at -80 °C until measurement with liquid chromatography with tandem mass spectrometry (LC–MS/MS).

### MRM measurement of NAD^+^, MNM, and NR with LC–MS/MS

The LC–MS/MS system was comprised of an HPLC system (ExionLC AD, AB SCIEX) coupled with a QTRAP6500 + mass spectrometer (AB Sciex) in electrospray ionization mode. Four microliters of the sample extract were injected onto an HILIC column (Atlantis Hilic, 4.6 mm × 50 mm, 5 μm, Waters) at 50 °C using a 7-min solvent gradient employing 10 mM of ammonium formate containing 0.05% of formic acid in water (mobile phase A) and acetonitrile (mobile phase B). Additional LC settings were as follows: 80% B in 1 min; 80 to 40% B in 2 min; 40% B in 1.5 min; 80% B in 2.5 min at a flow rate of 1.0 mL/min. MS settings for LC–MS/MS mode were as follows: curtain gas, 30; ion spray voltage, 5500 V; temperature, 400 °C; ion source gas 1, 50 psi; ion source gas 2, 60 psi; collision gas, 9 psi; entrance potential, 10 V. NAD^+^, NMN, and NR were identified and quantified using multiple reaction monitoring (MRM) in positive ion mode. MS settings for each target ion are shown in the Supplemental Table 2. Quantification was carried out using MultiQuant software (AB SCIEX) with stable isotope-labeled internal standards and calibration curves.

### Statistical analyses

ANOVA was used to determine whether there were any differences between the groups. Significance in differences was determined using the post hoc test at *p* < 0.05. Pearson's linear correlation of the metabolites and age of the 99 patients studied in the present study were also analyzed. Paired t tests were applied to test for significant differences between the Int. and POD30 samples. JASP (version 0.16.0.0) and R software with multcomp package were used for these analyses. Differences in sex for each metabolite were tested by t-test, with matching propensity scores for age and cancer type of 58 subjects (29 females and 29 males) using EZR software. All statistical analyses were performed for males and females, respectively, and no sex differences were found, so the data are presented without distinguishing between the sexes. In evaluation of the association between NAD^+^-related factors and perioperative clinical factors, continuous variables were expressed as medians (ranges) and analyzed using nonparametric methods for non-normally distributed data (Mann–Whitney U-test). Categorical variables were reported as numbers (percentages) and analyzed using the chi-squared test or Fisher’s exact test, as appropriate. A difference was considered to be significant for values of *P* < 0.05. The statistical analyses were performed using IBM SPSS Statistics for Windows, Version 26.0 (IBM Corp, Armonk, NY, USA).

### Consent for publication

 Consent for publication was obtained from each patient before enrollment.

### Supplementary Information

Supplementary Information 1.

Supplementary Information 2.

## Data Availability

The data generated or analyzed during this study are included in this published article and its supplementary information files.

## Abbreviations

Adm: Admission
BMI: Body mass index
Int: Initial intake medical examination
LC–MS/MS: Liquid chromatography with tandem mass spectrometry
MCV: Mean corpuscular volume
NAD^+^: Nicotinamide adenine dinucleotide
NAMPT: Nicotinamide phosphoribosyl transferase
NMN: Nicotinamide mononucleotide
NR: Nicotinamide riboside chloride
PARP: Poly (adenosine diphosphate-ribose) polymerases
POD1: The day after surgery
POD7: One week after surgery
POD30: One month after surgery
RBC: Red blood cell
SIRT: Sirtuin
