# Supplementary material for: Novel insight into nicotinamide adenine dinucleotide and related metabolites in cancer patients undergoing surgery
Source: Sci Rep. 2024 Jul 17;14:16557. doi: 10.1038/s41598-024-66004-1 (PMC11254928; doi:10.1038/s41598-024-66004-1)
Supplement: Supplementary file 1 — Supplementary Information 1. [file 41598_2024_66004_MOESM1_ESM.pdf]

Novel insight into nicotinamide adenine dinucleotide and related metabolites in cancer patients undergoing surgery

Hiroaki Fujita, Taiichi Wakiya, Yota Tatara, Keinosuke Ishido, Yoshiyuki Sakamoto, Norihisa Kimura, Hajime Morohashi, Takuya Miura, Takahiro Muroya, Harue Akasaka, Hiroshi Yokoyama, Taishu Kanda, Shunsuke Kubota, Aika Ichisawa, Kenta Ogasawara, Daisuke Kuwata, Yoshiya Takahashi, Akie Nakamura, Keisuke Yamazaki, Takahiro Yamada, Ryo Matsuyama, Masanobu Kanou, Kei Yamana, Ken Itoh, Kenichi Hakamada.

Supplementary information

Supplemental Content 1: Supplemental Figure 1, Comparison between females and males for NAD+, NMN, and NR.

Supplemental Content 2: Supplemental Figure 2, Perioperative line plot of NAD+ metabolites for each patient by cancer type.

Supplemental Content 3: Supplemental Figure 3, Association between NAD+ metabolites and preoperative chemotherapy.

Supplemental Content 4: Supplemental Figure 4, Association between NAD+ and operative time.

Supplemental Content 5: Supplemental Figure 5, Association between NAD+ and intraoperative bleeding.

Supplemental Content 6: Supplemental Figure 6, Analysis of association between NAD+ and postoperative complications.

Supplemental Content 7: Supplemental Information 1, Exclusion Criteria.

Supplemental Content 8: Supplemental Table 1, Correlation analysis of age, RBC volume-corrected NAD, NMN, and NR from initial medical examination samples.

Supplemental Content 9: Supplemental Table 2, MRM transitions and MS parameters for NAD+, NMN, and NR measurement.

Supplemental Content 10: Supplemental Information 2, Raw data.

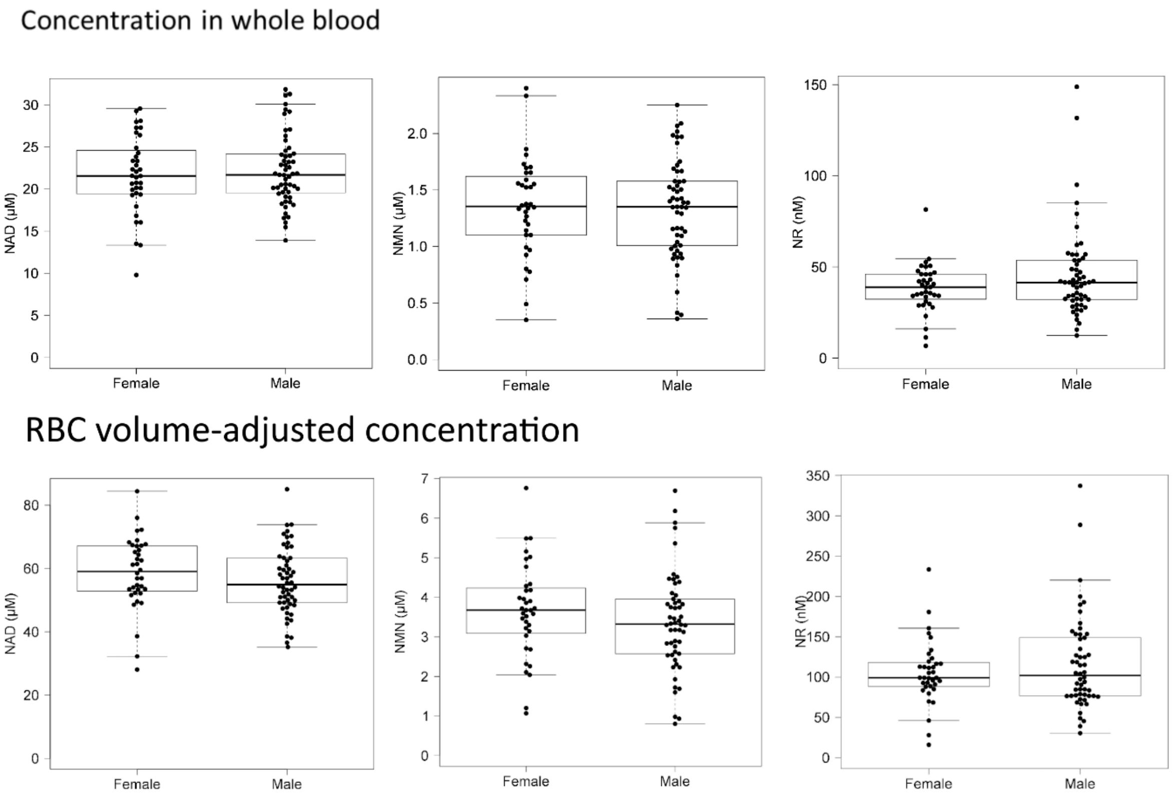

Supplemental Fig. S1

Comparison between females and males for NAD^+, NMN, and NR. The quantified whole blood NAD+-metabolite values were represented as concentrations adjusted for RBC volume.

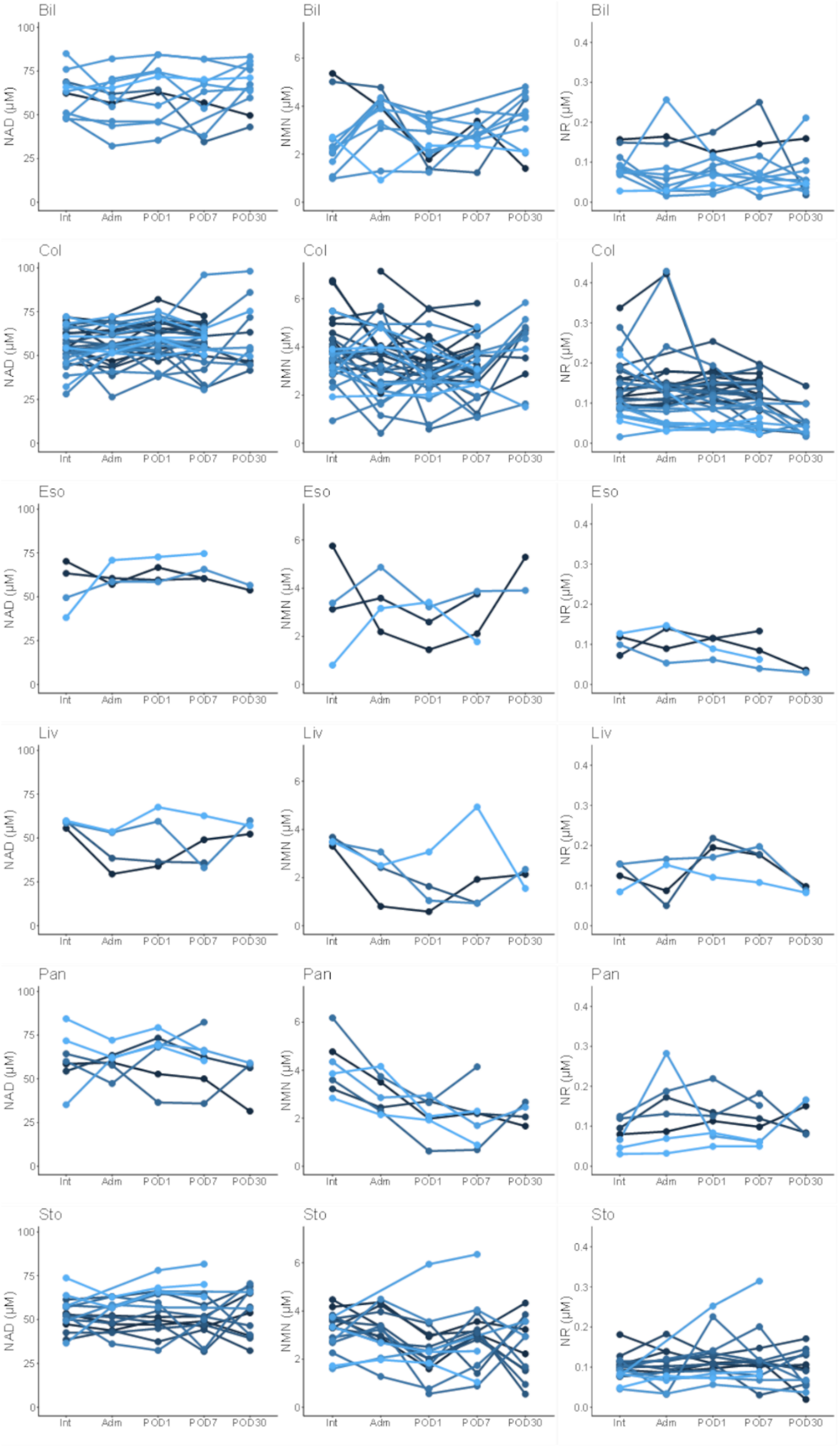

Supplemental Fig. S2

Perioperative line plot of NAD+ metabolites for each patient by cancer type.

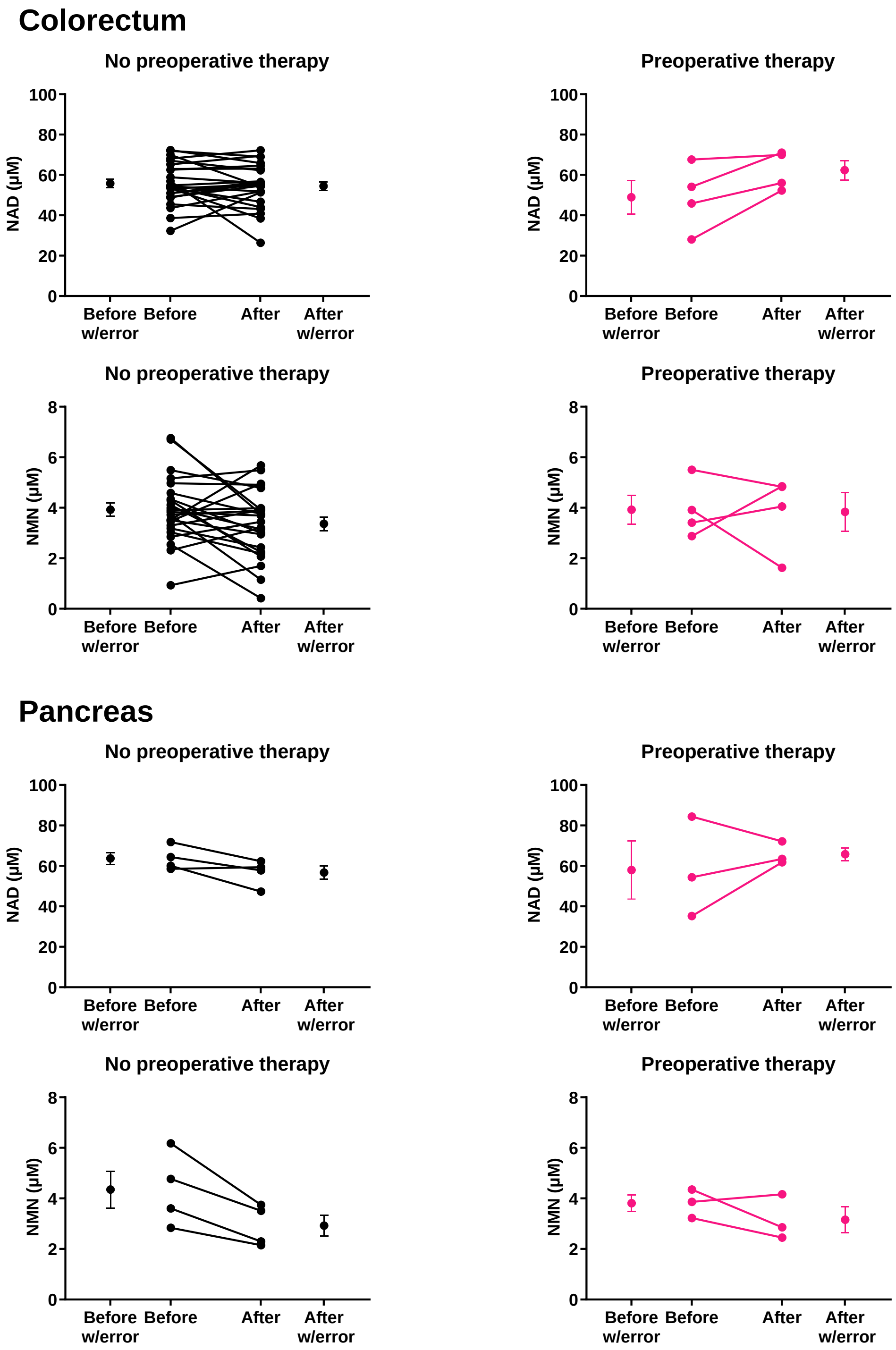

Supplemental Fig. S3

Association between NAD+ metabolites and preoperative chemotherapy. Each measurement was expressed as an RBC volume-corrected concentration.

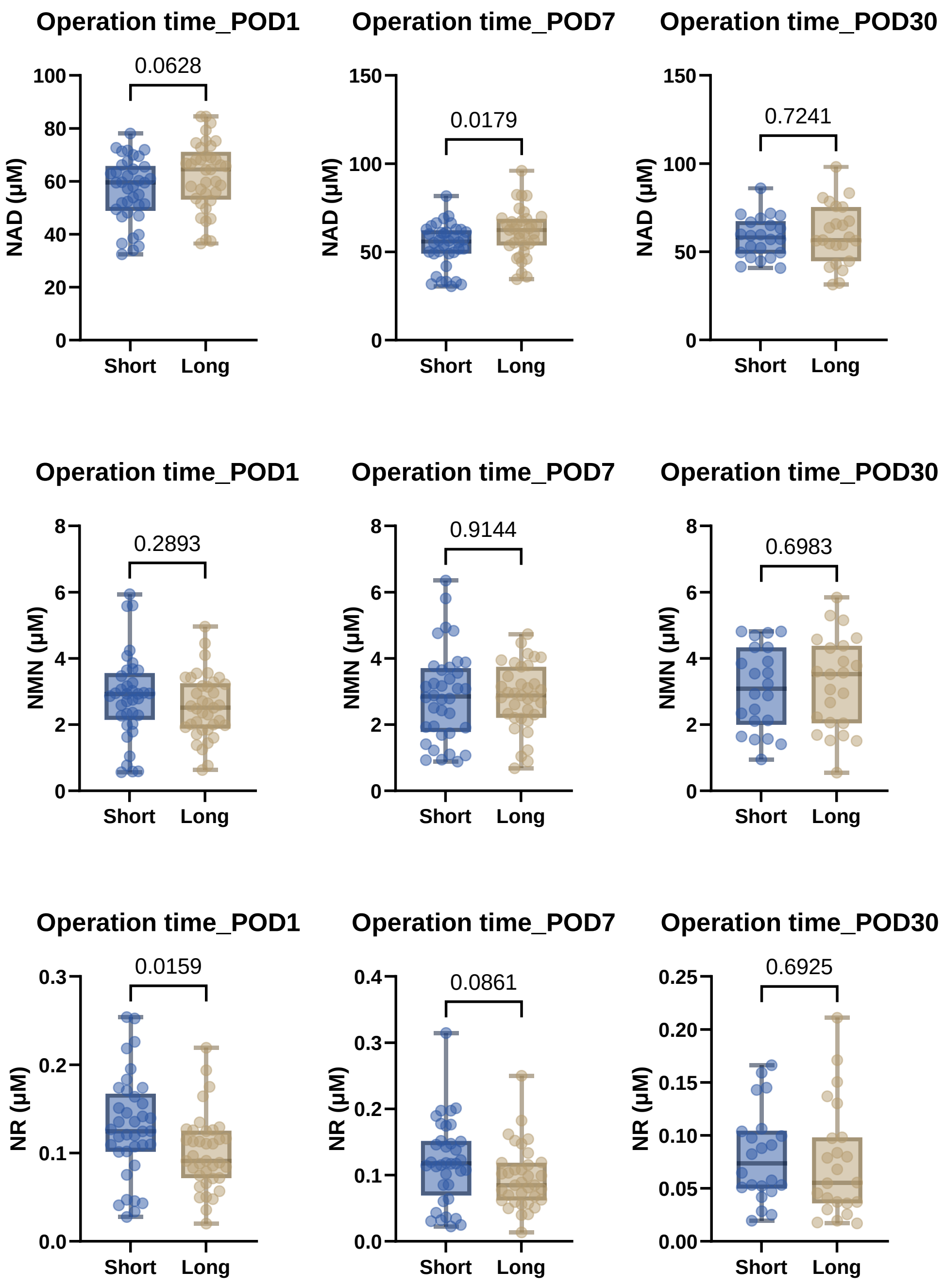

Supplemental Fig. S4

Association between NAD$^+$ and operative time. Measurements stratified by median operative time were represented in box plots. Each measurement was represented as an RBC volume-corrected concentration. POD1, the day after surgery; POD7, one week after surgery; POD30, one month after surgery.

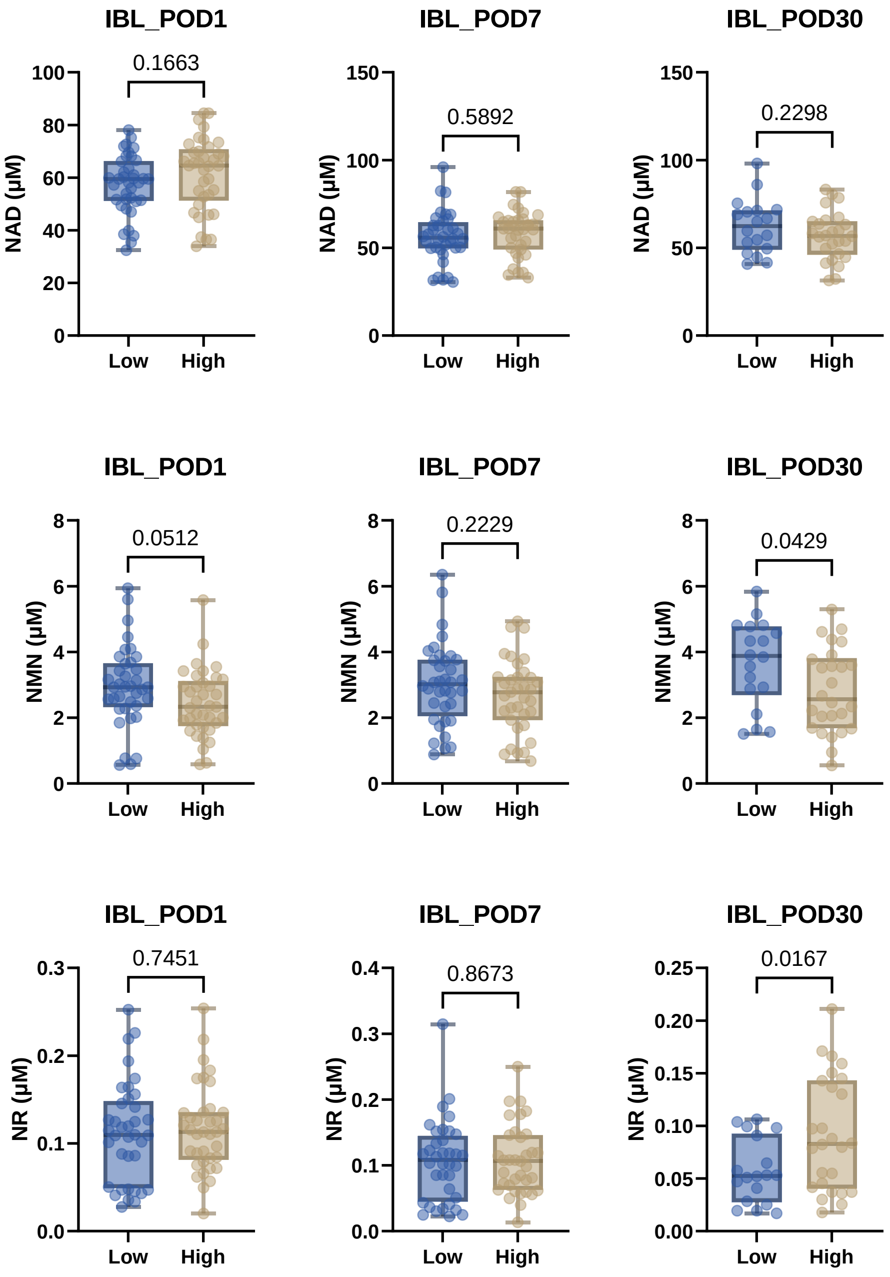

Supplemental Fig. S5

Association between NAD^+ and intraoperative bleeding. Measurements stratified by median intraoperative bleeding were represented in box-plots. Each measurement was represented as an RBC volume-corrected concentration. IBL, intraoperative bleeding; POD1, the day after surgery; POD7, one week after surgery; POD30, one month after surgery.

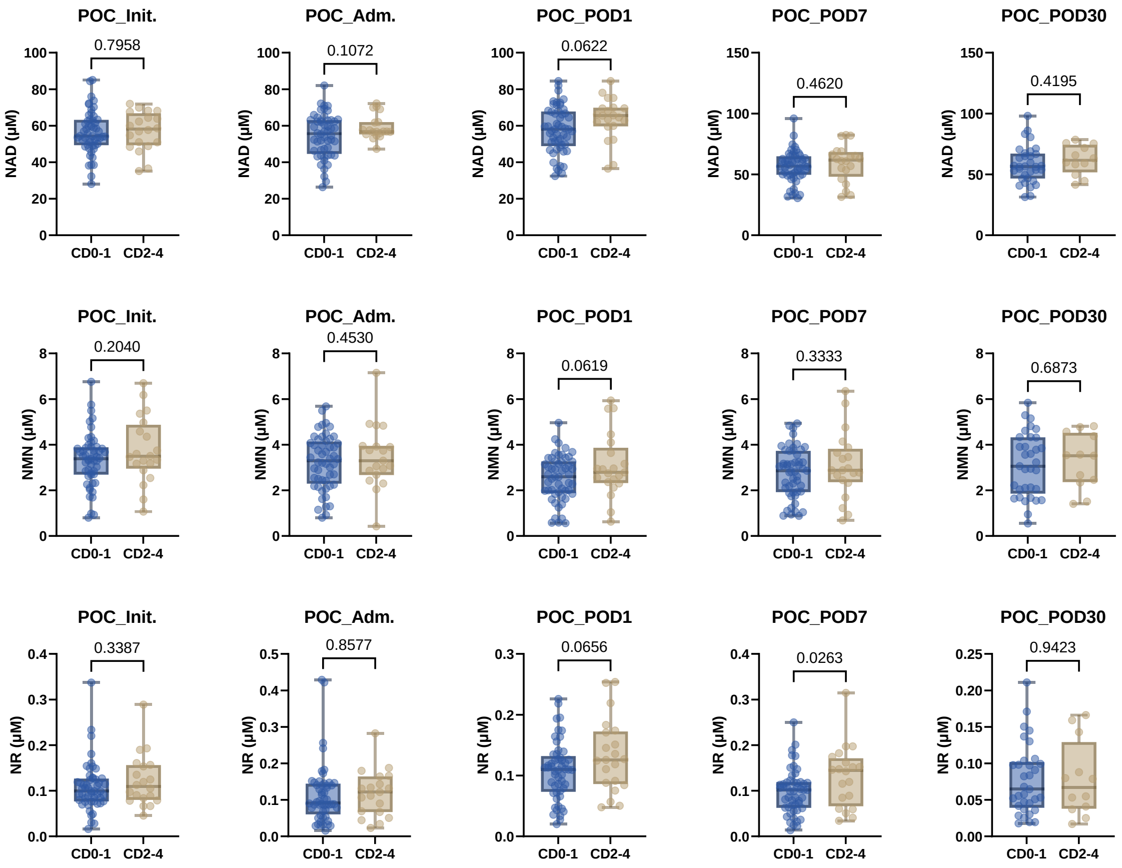

Supplemental Fig. S6

Analysis of association between NAD^+ and postoperative complications. Measurements stratified by no or minor complications (CD0-1) and moderate to severe complications (CD2-4) were represented in box plots. Each measurement was represented as a RBC volume-corrected concentrations. Adm., admission; Int., initial intake medical examination; POD1, the day after surgery; POD7, one week after surgery; POD30, one month after surgery.

Supplemental Information 1. Exclusion Criteria

1. Did not wish to participate in the study.
2. Deemed unsafe for the trial by a participating physician*

*Physicians were tasked with determining whether or not a patient was inappropriate for this trail based on safety reasons.

Supplemental Table 1. Correlation analysis of age, RBC volume-corrected NAD+, NMN, and NR from initial medical examination samples.

	95% CI			
	Lower	Upper	r	p value
NAD-NMN	0.043	0.431	0.247	0.018
NAD-NR	-0.195	0.217	0.012	0.913
NMN-NR	-0.037	0.364	0.170	0.106
NAD-Age	0.016	0.409	0.222	0.035
NMN-Age	0.022	0.414	0.227	0.030
NR-Age	-0.133	0.276	0.075	0.482

CI, confidence interval; NAD, nicotinamide adenine dinucleotide; NMN, nicotinamide mononucleotide; NR, nicotinamide riboside chloride.

Supplemental Table 2. MRM transitions and MS parameters for NAD^+, NMN, and NR measurement.

	Q1 (*m/z*)	Q3 (*m/z*)	Declustering potential (V)	Entrance potential (V)	Collision energy (V)	Collision cell exit potential (V)
NAD^+	664.06	136.10	101	10	117	14
NAD^+-d4	668.20	136.10	101	10	117	14
NMN	335.10	123.10	140	10	19	20
NMN-d4	339.14	126.90	140	10	19	20
NR	255.17	123.00	66	10	29	18
NR-d4	259.17	127.00	66	10	29	18

NAD^+, nicotinamide adenine dinucleotide; NMN, nicotinamide mononucleotide; NR, nicotinamide riboside chloride.
